# Knowledge, attitudes, and practices regarding constipation among patients with type 2 diabetes mellitus: a structural equation modeling analysis

**DOI:** 10.3389/fpubh.2026.1728483

**Published:** 2026-03-10

**Authors:** Qi Wang, Jin He, Dongzhi Zhang, Jing Yu, Min Tian, Qi Zhang, Sheng Zhou, Min Meng

**Affiliations:** 1 Medical Affairs Department, Gansu Provincial Hospital, Lanzhou, Gansu, China; 2 Party Committee Office, Gansu Provincial Hospital, Lanzhou, Gansu, China; 3 Department of Gerontology, Gansu Provincial Hospital, Lanzhou, Gansu, China; 4 Department of Radiology, Gansu Provincial Hospital, Lanzhou, Gansu, China

**Keywords:** constipation, cross-sectional studies, knowledge, attitudes, practice, structural equation modeling, type 2 diabetes mellitus

## Abstract

**Background:**

The present study aims to evaluate the knowledge, attitudes, and practices (KAP) related to constipation among patients diagnosed with type 2 diabetes mellitus (T2DM).

**Methods:**

This cross-sectional study was conducted at Gansu Provincial Hospital between October 2024 and May 2025. It aimed to assess the KAP regarding constipation among patients with type 2 diabetes mellitus through a questionnaire. Furthermore, structural equation modeling (SEM) was employed to analyze the relationships among the different KAP components.

**Results:**

A total of 364 complete and valid questionnaires were included in the final analysis. The median age of the patients was 55 (47, 62) years, with 64.3% being male, and 41.2% having attained at least an associate's or bachelor's degree. The median (interquartile range) scores for knowledge, attitude, and practice were 6 (3.25, 9), 37 (35, 42), and 30 (27, 33), respectively (possible ranges: 0–15, 10–50, and 10–50). SEM results revealed that knowledge had a direct effect on both attitudes (β = 0.298, *P* = 0.004) and practices (β = 0.235, *P* = 0.019). Attitudes also had a direct effect on practices (β = −0.188, *P* = 0.009). Furthermore, knowledge indirectly influenced practices through attitudes (β = −0.056, *P* = 0.005).

**Conclusions:**

Patients diagnosed with T2DM generally exhibited insufficient knowledge of constipation. Although they typically held positive attitudes, there was a considerable lack in their self-management practices for constipation, highlighting a significant knowledge-practice gap. This study suggests that healthcare providers should prioritize implementing targeted, evidence-based educational interventions to enhance patients' knowledge and translate positive attitudes into effective self-care behaviors.

## Background

Type 2 diabetes mellitus (T2DM) represents a chronic metabolic disorder characterized by insulin resistance, impaired insulin secretion, and persistent hyperglycemia (1). The global burden of this condition is substantial, with the World Health Organization reporting that T2DM accounts for more than 90% of all diabetes cases worldwide (2). Current epidemiological data indicate that approximately 537 million adults were affected in 2021, with projections suggesting this figure will escalate to 783 million by 2045 (3). Notably, the prevalence in China has reached 12.4% among adults as of 2018, surpassing the global average (4). The clinical significance of T2DM extends beyond glycemic disturbances, as it frequently precipitates devastating complications including cardiovascular disease, nephropathy, retinopathy, and neuropathy, collectively contributing to substantial morbidity and diminished quality of life (5).

Constipation constitutes a prevalent gastrointestinal disorder defined by infrequent bowel movements or difficulty with defecation, typically diagnosed according to Rome IV criteria (6). The prevalence of constipation among T2DM patients demonstrates a striking disparity, ranging from 30 to 60%, which markedly exceeds the approximately 14% prevalence observed in the general population (7, 8). This elevated risk primarily stems from diabetic autonomic neuropathy. In addition, multiple contributing factors further compound this association, including delayed gastric emptying, sedentary lifestyle patterns, inadequate fiber, and fluid consumption, adverse medication effects, and disrupted gut microbiota homeostasis (9). Furthermore, constipation may compromise glycemic management through impaired drug absorption and reduced dietary adherence. Despite its clinical relevance, constipation remains significantly underaddressed in diabetes care protocols, with numerous patients either dismissing symptoms or resorting to prolonged, unsupervised laxative use, potentially exacerbating clinical outcomes (10). These observations highlight the critical need for systematic educational initiatives and behavior-oriented interventions to enhance constipation management within comprehensive T2DM care.

The Knowledge, Attitude, and Practice (KAP) framework serves as a validated diagnostic research instrument that elucidates individuals' comprehension, perceptions, and behavioral patterns concerning specific health conditions (11, 12). Within health literacy research, the KAP model operates on the theoretical premise that knowledge acquisition influences attitudinal formation, which subsequently determines behavioral practices (13). This methodological approach has demonstrated extensive utility in chronic disease investigations, particularly diabetes research, where it has been employed to evaluate patient engagement in critical self-management activities including blood glucose monitoring, dietary adherence, and preventive foot care (14, 15). The majority of existing KAP investigations have concentrated on well-established diabetic complications such as diabetic foot ulceration or general diabetes self-management, consistently revealing that patients frequently exhibit suboptimal practices despite possessing adequate theoretical knowledge (16, 17). However, the application of this framework to gastrointestinal complications—particularly constipation, which represents a highly prevalent yet consistently underrecognized complication in T2DM—remains notably limited.

To bridge this knowledge gap, the present cross-sectional investigation systematically examines the knowledge, attitudes, and practices of patients with T2DM regarding constipation management. By employing a validated behavioral assessment framework, this study seeks to generate robust, generalizable, and patient-centered evidence that will inform the development of targeted educational interventions and enhance constipation management protocols within comprehensive diabetes care.

## Methods

### Study design and participants

This cross-sectional study was conducted between October 2024 and May 2025 at Gansu Provincial Hospital. Patients with T2DM were invited to complete questionnaires. The inclusion criteria were: (1) meeting the diagnostic criteria for type 2 diabetes mellitus according to the American Diabetes Association (ADA) guidelines. Exclusion criteria were: (1) patients with severe primary diseases, including advanced or decompensated cardiac dysfunction, severe hepatic impairment, or advanced renal dysfunction requiring intensive medical management, as well as hematopoietic system disorders or psychiatric disorders; (2) individuals lacking legal capacity or insight, or those unable to understand or complete the questionnaire. This study received an ethics exemption from the Ethics Committee of Gansu Provincial Hospital and obtained informed consent from all participants prior to the start of the research. Glycemic control status was classified according to ADA recommendations. Patients with glycated hemoglobin (HbA1c) levels < 7.0% were defined as having controlled diabetes, whereas those with HbA1c levels ≥7.0% were classified as uncontrolled. Glycemic control treatment was provided according to routine clinical practice and current guideline recommendations, including lifestyle modification and pharmacological therapy such as oral hypoglycemic agents and/or insulin. Information on antidiabetic treatment was collected from medical records; however, treatment regimens were not used as stratification variables in the main statistical analyses.

### Sample size estimation

The minimum required sample size for this cross-sectional study was calculated using the single population proportion formula. In this methodology, *n* equals (*Z* squared multiplied by *p* multiplied by 1 minus *p*) divided by *d* squared, where *Z* represents the standard normal deviate corresponding to a 95 percent confidence level of 1.96, *p* denotes the estimated proportion of knowledge, attitude, or practice at 0.5 to ensure maximum sample variability, and *d* signifies the allowable margin of error set at 0.05. According to this formula, the theoretical minimum sample size required to ensure the statistical representativeness of the findings was determined to be 384 participants. This calculation provides the necessary framework for evaluating the precision of the estimated parameters within the target population.

### Questionnaire design

The questionnaire was designed with reference to relevant clinical guidelines (18). Upon completion of the initial draft, three experts with associate senior professional titles or above from the departments of geriatrics, endocrinology, and traditional Chinese medicine were invited to review its content. Adjustments were then made to ensure the questions were easily understood by participants. Subsequently, a small-scale pilot study was conducted, yielding 21 valid questionnaires. Reliability analysis indicated that the Cronbach's α coefficient was 0.925, demonstrating the questionnaire's good internal consistency. Exploratory factor analysis (EFA) was conducted to examine the underlying factor structure of the KAP items. Sampling adequacy was assessed using the Kaiser–Meyer–Olkin (KMO) measure and Bartlett's test of sphericity. Factors were extracted using principal axis factoring with oblique rotation. Confirmatory factor analysis (CFA) was then performed using Amos to evaluate the measurement model, with model fit assessed by CMIN/DF, RMSEA, IFI, TLI, and CFI. Negatively worded items were reverse-coded prior to factor analyses.

The final version of the questionnaire included four parts, with a total of 48 items (Supplementary Appendix). The basic information section contained 14 items. The knowledge section consisted of 13 items, using three question types: true/false (one point for correct answers, 0 for incorrect or uncertain), single-choice (one point for correct answers, 0 for incorrect or uncertain), and multiple-choice (two points if all selected options were correct, one point if partially correct, and 0 for incorrect). The total possible score for the knowledge section ranged from 0 to 15. The attitude section included 10 items, assessed using a five-point Likert scale from “Strongly Agree” (five points) to “Strongly Disagree” (one point), depending on the direction of the statement. The possible score ranged from 10 to 50. The practice section also had 10 items, rated on a five-point Likert scale from “Always” (five points) to “Never” (one point), with a total score range of 10 to 50. According to the proportion of the maximum possible score, KAP levels were categorized as insufficient (< 60% of the total score) and sufficient (≥60% of the total score). This classification approach has been widely applied in KAP-based research to facilitate interpretation of overall performance.

### Data collection and quality control

Questionnaires were distributed via the Wenjuanxing platform (www.wjx.cn). To ensure data quality and integrity, each IP address was limited to one submission, and all questionnaire items were set as mandatory. The research team manually reviewed the collected questionnaires to check for completeness, internal consistency, and the rationality of responses. To further ensure data reliability, questionnaires were excluded if answers were deemed illogical, or if any section of the KAP scale showed identical responses across all items.

### Statistical analyses

All statistical analyses were performed using SPSS version 27.0 (IBM Corporation, Armonk, NY, USA) and AMOS version 26.0 (IBM Corporation, Armonk, NY, USA). Continuous variables were described as means ± standard deviations or medians (quartiles), and categorical variables were described as frequencies and percentages. In the KAP scale, the original form of each item in the Knowledge dimension was a categorical variable. Before the structural equation model analysis, multiple items of the Knowledge dimension were summed to calculate the comprehensive Knowledge score of each subject. This composite score was included in subsequent analyses as a continuous variable, a treatment that is widely adopted in KAP research and is applicable to structural equation models based on the assumption of continuous variables. Between-group comparisons were conducted using nonparametric statistical methods, including the Mann–Whitney *U*-test for two-group comparisons and the Kruskal–Wallis *H*-test for multiple-group comparisons. Spearman's rank correlation analysis was applied to examine associations among KAP scores. The structural equation model (SEM) was used to examine the relationships among knowledge, attitude, and practice. Model goodness of fit was evaluated using the comparative fit index (CFI), Tucker–Lewis index (TLI), and root mean square error of approximation (RMSEA). The mediation analysis was conducted within the SEM framework, with the attitude dimension specified as the mediating variable, the knowledge dimension as the independent variable, and the practice dimension as the dependent variable. Direct and indirect effects were estimated simultaneously. The significance of mediation effects was assessed using bootstrap resampling with 5,000 iterations, and 95% confidence intervals were calculated. Mediation effects were considered statistically significant when the confidence interval did not include zero. A two-sided *P* value < 0.05 was considered statistically significant.

Adopting the KAP framework, SEM was employed to examine the mediating role of attitudes between knowledge and practices. Both direct and indirect path coefficients were calculated. Model fit was assessed using the Root Mean Square Error of Approximation (RMSEA), Standardized Root Mean Square Residual (SRMR), Incremental Fit Index (IFI), Tucker-Lewis Index (TLI), and Comparative Fit Index (CFI). An RMSEA value less than 0.08, and IFI, TLI, and CFI values greater than 0.90 were generally considered to indicate an acceptable model fit. A two-sided *P*-value less than 0.05 was considered statistically significant.

## Results

A total of 364 valid questionnaires were finally included. The Cronbach's α coefficient for the formal survey was 0.855, indicating good internal consistency of the questionnaire. Exploratory factor analysis supported factorability of the KAP items (KMO = 0.826; Bartlett' *s*-test of sphericity: χ^2^ = 4,254.331, df = 465, *P* < 0.001), and seven factors were extracted using principal axis factoring with oblique rotation (Supplementary Table S1). Confirmatory factor analysis further indicated an acceptable measurement model fit (CMIN/DF = 2.427; RMSEA = 0.063; IFI = 0.849; TLI = 0.831; CFI = 0.848), with all standardized factor loadings statistically significant (*P* < 0.05); (Supplementary Table S2, Supplementary Figure S1).

### Baseline characteristics

The median age of participants was 55 (47, 62) years. Among them, 64.3% were male and 41.2% had attained an associate's or bachelor's degree or higher. The median (interquartile range) scores for knowledge, attitude, and practice were 6 (3.25, 9), 37 (35, 42), and 30 (27, 33), respectively. Significant differences in knowledge scores were observed across educational level [Bachelor or above: 7 (4, 9) vs. Primary school or below: 6 (0.5, 8)], vegetarian status [Non-vegetarian: 7 (4, 9) vs. Vegetarian: 4 (2, 7)], duration of diabetes [≥20 years: 3.5 (2, 6.25) vs. ≤ 5 years: 7 (4, 9)], and glycemic control status defined by HbA1c level [Uncontrolled: 7 (4, 9) vs. Controlled: 6 (3, 8)] (all *P* < 0.05). Attitude scores differed significantly by educational level [Bachelor or above: 38.5 (36, 42) vs. Primary school or below: 36 (33, 42), *P* = 0.004]. Practice scores were significantly associated with smoking status [Non-smokers: 30 (27, 33) vs. Smokers: 29 (26, 32), *P* = 0.011], glycemic control [Controlled: 31 (28, 34) vs. Uncontrolled: 29 (26, 32), *P* < 0.001], and bowel symptom status [None: 31 (27, 33) vs. fewer than three bowel movements per week: 27 (25, 32), *P* = 0.003]; (Table 1).

**Table 1:** Demographic characteristics and KAP scores.

**Variable**	***N* (%)**	**Knowledge, median, IQ range**	***P***	**Attitude, median, IQ range**	***P***	**Practice, median, IQ range**	***P***
***N*** **=** **364**		6 (3.25, 9)		37 (35, 42)		30 (27, 33)	
**Total score**			
Age	55 (47, 62)						
Gender			0.660		0.773		0.903
Male	234 (64.29%)	6 (3, 9)		37 (35, 42)		30 (26.75, 33)	
Female	130 (35.71%)	7 (4, 8)		37 (35, 42)		30 (26.75, 33)	
Weight	69.25 (60.25, 80)						
Height	168 (162, 172)						
BMI			0.845		0.831		0.057
Normal	197 (54.12%)	6 (4, 8)		37 (34.5, 42)		30 (26, 34)	
Overweight	122 (33.52%)	7 (3, 9)		37 (35, 41)		30 (27, 33)	
Obesity	45 (12.36%)	6 (3, 9)		38 (35, 42.5)		28 (26.5, 30.5)	
Monthly income, CYN			0.070		0.064		0.329
< 2,000	105 (28.85%)	6 (3, 8)		37 (35, 42)		29 (26, 33)	
2,001–5,000	175 (48.08%)	7 (4, 9)		36 (35, 41)		30 (27, 33)	
>5,001	84 (23.08%)	7 (3, 9)		39 (35, 43)		30 (28, 34)	
Education			0.021		0.004		0.127
Primary school or below	57 (15.66%)	6 (0.5, 8)		36 (33, 42)		29 (26, 33)	
Junior high school	66 (18.13%)	6 (3, 9)		36 (34, 39.25)		28 (26, 32)	
Senior high school	91 (25.00%)	7 (4, 9)		37 (34, 42)		30 (28, 33)	
Bachelor or above	150 (41.21%)	7 (4, 9)		38.5 (36, 42)		31 (27, 33)	
Marital status			0.799		0.158		0.688
Married	330 (90.66%)	6 (3.75, 9)		37 (35, 42)		30 (27, 33)	
Others	34 (9.34%)	6.5 (3, 9)		39 (35.75, 43)		30 (26, 32.25)	
Vegetarian			< 0.001		0.942		0.778
Yes	75 (20.60%)	4 (2, 7)		38 (34, 42)		30 (27, 33)	
No	289 (79.40%)	7 (4, 9)		37 (35, 42)		30 (26, 33)	
Duration			0.047		0.358		0.545
≤ 5 years	200 (54.95%)	7 (4, 9)		37 (35, 42)		30 (26.25, 33)	
6–10 years	73 (20.05%)	6 (4, 9)		36 (34, 41.5)		30 (26, 33.5)	
11–20 years	65 (17.86%)	7 (4, 9)		38 (35.5, 42)		30 (26, 34)	
≥ 20 years	26 (7.14%)	3.5 (2, 6.25)		37 (31.75, 40.5)		30.5 (27.75, 34.25)	
Blood glucose level is under control			< 0.001		0.243		< 0.001
Yes	164 (45.05%)	6 (3, 8)		37 (34, 42)		31 (28, 34)	
No	200 (54.95%)	7 (4, 9)		38 (35, 42)		29 (26, 32)	
Smoking			0.851		0.614		0.011
Yes	107 (45.05%)	6 (4, 9)		38 (35, 42)		29 (26, 32)	
No	257 (70.60%)	6 (3, 9)		37 (35, 42)		30 (27, 33)	
Drinking			0.496		0.173		0.109
Yes	98 (26.92%)	6 (3, 9)		38 (35, 42)		29 (26, 32.25)	
No	266 (73.08%)	7 (3.75, 9)		37 (35, 42)		30 (27, 33)	
Symptom			0.059		0.931		0.003
Straining during bowel movements, hard stools, a feeling of incomplete evacuation, sensation of anorectal obstruction, or needing manual assistance	83 (22.80%)	6 (3, 8)		38 34, 42)		29 (26, 33)	
Fewer than 3 spontaneous bowel movements per week	41 (11.26%)	7 (5, 8)		36 (35, 41.5)		27 (25, 32)	
Diagnosed with constipation by a physician	9 (2.47%)	6 (4, 8.5)		36 (33, 43.5)		29 (26, 33)	
None of the above	231 (63.46%)	7 (3, 9)		37 (35, 42)		31 (27, 33)	

### Distribution of KAP

As shown in the distribution of knowledge dimension, regarding medication risks, only 12.64% correctly identified insulin as the drug least likely to cause constipation (K12), while 80.22% expressed uncertainty—the highest uncertainty rate across all questions. When it comes to non-recommended interventions, 10.44% erroneously believed increasing dietary fiber is inappropriate for constipation relief (K10), and 63.74% correctly rejected laxative misuse though 21.15% remained unsure. Talking about pathophysiology misconceptions, 16.48% incorrectly claimed constipation relates solely to diet and not diabetes (K5), with 31.04% uncertain. Additionally, 21.70% falsely asserted diabetics should not increase fiber intake (K2). Meanwhile, 25.27% were uncertain about multifactorial causes of diabetic constipation (K11) (Supplementary Table S3).

Responses to the attitude dimension showed that 21.43% strongly agreed and 42.58% agreed that persistent constipation related to diabetes makes them feel depressed. (A9), as well as 8.79% strongly agreed and 21.15% agreed that constipation is a difficult topic to talk about (A8). On the other hand, 4.95% very unwilling and 12.64% unwilling to try treatments such as acupuncture and other traditional therapies (A10); (Supplementary Table S4).

Responses to the practice dimension showed that 40.38% reported always using laxatives or traditional Chinese herbal purgative preparations (referred to as “heat-clearing medications”) on their own when experiencing constipation (P7), and 27.20% always delayed going to the restroom when they felt the urge to defecate (P5). In addition, 17.86% were unable to have regular bowel movements every day (P2), and 12.64 % were unable to ensure aerobic exercise at least three times a week (P3); (Supplementary Table S5).

### Correlations between KAP

Further correlation analysis revealed positive correlations between knowledge scores and attitude scores (*r* = 0.192, 95% CI: 0.088–0.292, *P* < 0.001), as well as between knowledge scores and practice scores (*r* = 0.142, 95% CI: 0.037–0.244, *P* = 0.007). In addition, attitude scores showed a borderline positive correlation with practice scores (*r* = 0.103, 95% CI: −0.003–0.206, *P* = 0.050); (Table 2).

**Table 2:** Correlation analysis.

**Dimension**	**Knowledge**	**Attitude**	**Practice**
Knowledge	1		
Attitude	0.192 (0.088–0.292), *P* < 0.001	1	
Practice	0.142 (0.037–0.244), *P* = 0.007	0.103 (−0.003–0.206), *P* = 0.050	1

### SEM analysis

**Figure 1:**
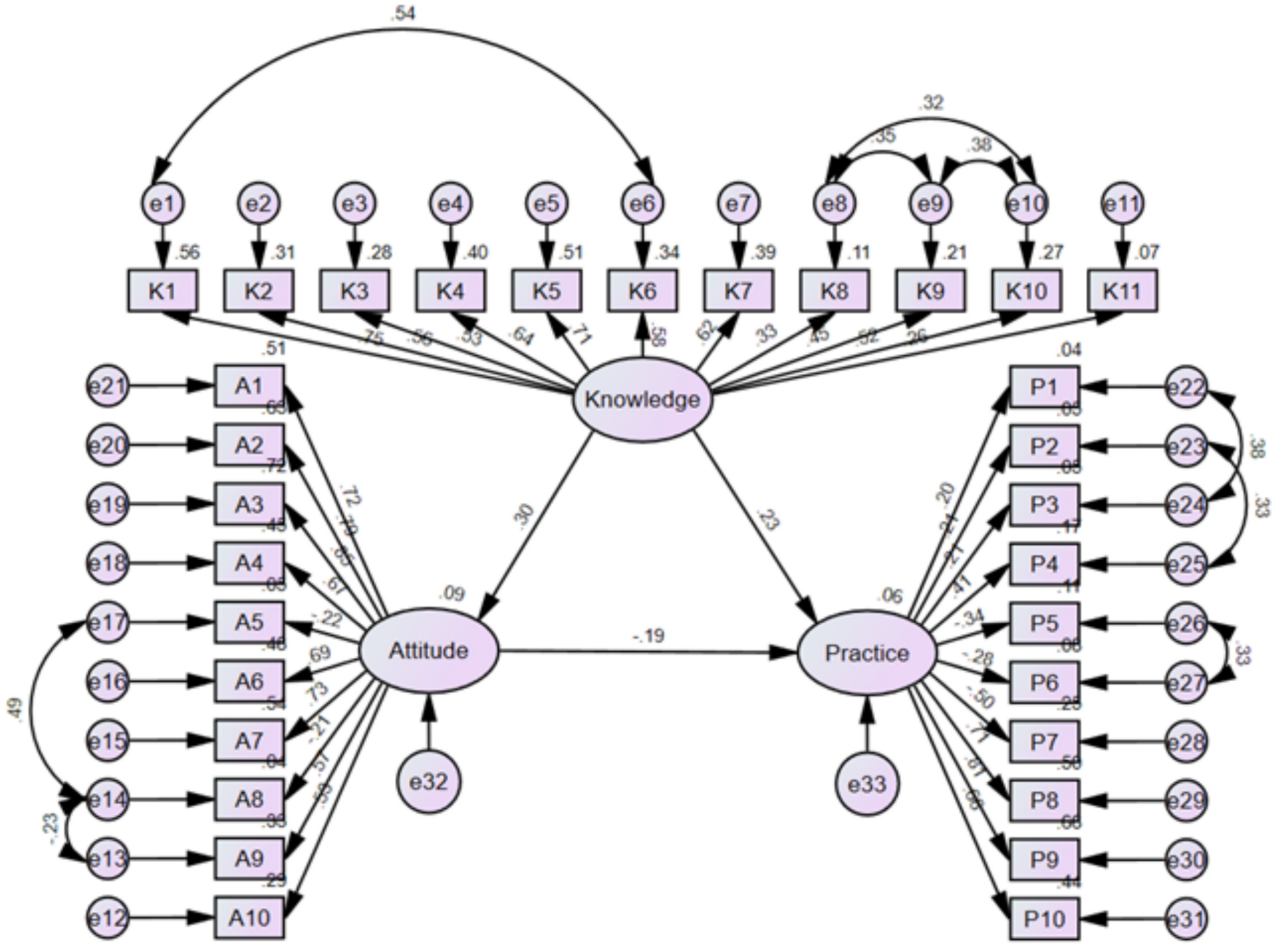
SEM model.

The fit of the SEM model demonstrated acceptable goodness-of-fit indices (CMIN/DF = 2.575; IFI = 0.832; TLI = 0.813; CFI = 0.831; RMSEA = 0.066; SRMR = 0.080), and detailed model fit statistics are presented in (Supplementary Table S6). The estimated total effects of knowledge and attitude on practice are summarized in (Supplementary Table S7). The results showed that knowledge had a significant direct effect on both attitude (β = 0.298, *P* = 0.004) and practice (β = 0.235, *P* = 0.019), while attitude also exerted a direct effect on practice (β = −0.188, *P* = 0.009). Furthermore, knowledge indirectly affected practice through attitude (β = −0.056, *P* = 0.005); (Table 3, Figure 1).

**Table 3:** SEM direct and indirect effects.

**Model paths**	**Standardized direct effects (95% CI)**	***P***	**Standardized indirect effects (95% CI)**	***P***
Knowledge → attitude	0.298 (0.177 to 0.448)	0.004		
Knowledge → practice	0.235 (0.094 to 0.387)	0.019	−0.056 (−0.148 to −0.016)	0.005
Attitude → practice	−0.188 (−0.410 to −0.055)	0.009		

## Discussion

The study findings indicate that patients with type 2 diabetes mellitus have limited knowledge of constipation management. While generally holding positive attitudes, their self-management practices are notably insufficient. These findings underscore the necessity for integrated, behavior-oriented educational interventions within diabetes care, which should aim not only to enhance patients' knowledge but also to actively support the translation of awareness into effective self-management practices.

Among patients with type 2 diabetes, knowledge related to the pathophysiology, risk factors, and management of constipation remains fragmented and, in some domains, markedly deficient. Notably, patients with a longer duration of diabetes showed lower knowledge scores, which may be partially explained by aging-related cognitive decline, reliance on outdated health information, and reduced participation in continuing diabetes education over time. In addition, long-term disease burden and self-management fatigue may further weaken patients' motivation to actively update health-related knowledge. In the current data, although a number of participants demonstrated awareness of fluid intake and dietary fiber as beneficial, there was substantial confusion regarding drug-induced constipation and when to seek clinical intervention. Such findings mirror trends observed in chronic illness populations where non-primary symptoms often fall outside the dominant educational focus, leading to underdeveloped cognitive frameworks for symptom recognition and response (19, 20). Even among those with higher educational attainment, incorrect responses and uncertainty in foundational knowledge items suggest that existing health education approaches may not have adequately addressed this comorbidity.

The SEM supports the conclusion that knowledge exerts a moderate influence on both attitudinal and behavioral domains, with a measurable direct path to reported practices. However, the magnitude of these effects does not suggest a linear progression from knowledge to behavior. This implies that while knowledge may provide an essential conceptual foundation, it is not by itself a reliable predictor of action in contexts where cultural perception, emotional framing, or normative beliefs dilute the impetus to act. Studies focused on gastrointestinal health in diabetic populations have similarly observed that knowledge about autonomic complications or lifestyle adjustment rarely suffices to catalyze behavioral change without parallel emotional or normative reinforcement (21, 22).

Attitudinal responses in the current sample appeared broadly favorable, yet they did not correspond with either consistent practice or clear behavioral intent. Although many respondents expressed willingness to change dietary habits or adopt non-pharmacological interventions when experiencing constipation, their reported practices often contradicted these positions. This divergence was particularly pronounced in areas such as physical activity, proactive clinical consultation, and adherence to bowel routines. The model-based analyses indicated that attitudinal variables had limited or even suppressive effects on practice. While such a pattern may seem counterintuitive, prior behavioral science literature has suggested that socially desirable responding or passive agreement in survey contexts can obscure underlying reluctance or resignation, especially in symptom domains considered sensitive or private (23, 24).

The distribution of practice-related items reinforces this tension between stated intention and actual behavior. For instance, only a minority of respondents reported regular consumption of fiber or sustained physical activity. In contrast, self-directed use of over-the-counter remedies was much more common. This suggests that many patients may be relying on symptomatic relief rather than preventive strategies, a pattern frequently observed in populations managing multiple chronic conditions with limited external support (25, 26). Interestingly, those who reported more frequent symptoms were not more likely to engage in constructive behaviors, nor did their knowledge levels appear consistently higher, indicating that experience alone does not suffice to modify patterns of action.

To address these interlocking gaps, interventions must extend beyond generic health education. At the community care level, brief and recurrent sessions focusing specifically on bowel health could be embedded into existing diabetes management programs. These sessions should avoid abstract instruction, instead emphasizing scenario-based learning and symptom-to-strategy mapping. For patients with low literacy or high symptom burden, practical tools such as illustrated behavior guides, symptom logs, and one-on-one coaching from trained nurses may foster gradual behavioral integration. Within clinical settings, gastrointestinal symptom screening should be integrated into routine diabetes follow-up visits, allowing for early detection and targeted advice. Primary care workflows can incorporate brief digital checklists that prompt clinicians to explore defecation-related concerns, especially in patients reporting dietary or mobility challenges (27, 28).

Such strategies require alignment across multiple levels of the care system. Clinical guidelines should acknowledge bowel health as a core domain of quality diabetes care rather than a peripheral concern. Institutional protocols might consider linking gastroenterological education to metabolic control outcomes, thereby incentivizing comprehensive consultations. At the patient interface, any effort to modify behavior should involve not only cognitive activation but also repeated behavioral rehearsal under supportive supervision. Evidence from adjacent fields, such as hypertension self-management, indicates that structured practice loops involving patient-led goal setting and routine feedback can outperform didactic instruction alone in sustaining behavior over time (29, 30).

This study has several limitations that should be acknowledged. First, the cross-sectional design precludes the ability to establish causal relationships among KAP. Second, the reliance on self-reported data may introduce recall and social desirability biases, potentially affecting the accuracy of responses. Third, the study was conducted at a single institution and may not fully represent the broader population of patients with T2DM, limiting the generalizability of the findings. Finally, the final number of participants included in the analysis was 364, which is slightly below the theoretically calculated minimum sample size of 384 based on the single population proportion formula. While this discrepancy might marginally increase the margin of error, the sample size remains well within the methodologically recommended range of 5 to 10 times the number of questionnaire items (31), thereby maintaining sufficient statistical reliability for the primary objectives of this KAP assessment.

In conclusion, patients diagnosed with T2DM demonstrated insufficient knowledge, generally positive attitudes, but suboptimal practices in managing constipation, highlighting a significant knowledge-practice gap. To enhance patient outcomes, clinical interventions should prioritize structured educational programs that not only improve knowledge but also actively translate positive attitudes into effective self-care behaviors.

## Data Availability

The original contributions presented in the study are included in the article/Supplementary material, further inquiries can be directed to the corresponding authors.

## Supplementary material

Supplementary Figure S1 Confirmatory factor analysis (CFA) measurement model of the KAP questionnaire.

Supplementary Table S1 Exploratory factor analysis factor loading matrix of the KAP questionnaire.

Supplementary Table S2 Model fit indices of confirmatory factor analysis.

Supplementary Table S3 Distribution of knowledge dimension responses.

Supplementary Table S4 Distribution of attitude dimension responses.

Supplementary Table S5 Distribution of practice dimension responses.

Supplementary Table S6 SEM model fit.

Supplementary Table S7 Estimated total effect coefficient.
