# Supplementary figures and images for: Knowledge, attitudes, and practices regarding constipation among patients with type 2 diabetes mellitus: a structural equation modeling analysis
Source: Front Public Health. 2026 Mar 10;14:1728483. doi: 10.3389/fpubh.2026.1728483 (PMC13008887; doi:10.3389/fpubh.2026.1728483)

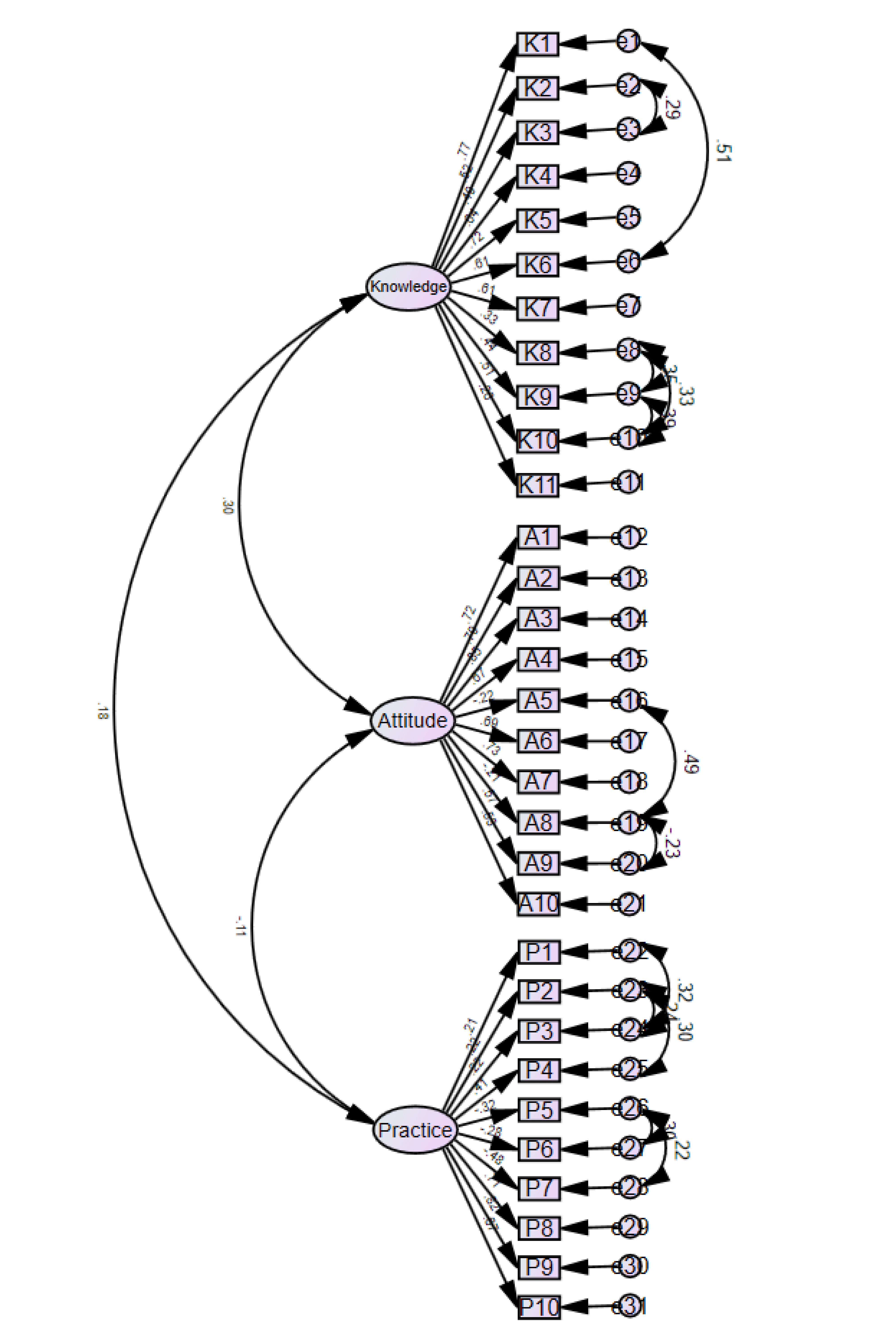

Supplement: Supplementary Figure S1 — Confirmatory factor analysis (CFA) measurement model of the KAP questionnaire. [file Image_1.tif]
