# Supplementary material for: Knowledge, attitudes, and practices regarding constipation among patients with type 2 diabetes mellitus: a structural equation modeling analysis
Source: Front Public Health. 2026 Mar 10;14:1728483. doi: 10.3389/fpubh.2026.1728483 (PMC13008887; doi:10.3389/fpubh.2026.1728483)
Supplement: Supplementary Table S1 — Exploratory factor analysis factor loading matrix of the KAP questionnaire. [file Data_Sheet_2.docx]

**Supplementary table S1. Exploratory factor analysis factor loading matrix of the KAP questionnaire**

| **Factor matrix^a^** | | | | | | | |
| --- | --- | --- | --- | --- | --- | --- | --- |
|  | Factor | | | | | | |
|  | 1 | 2 | 3 | 4 | 5 | 6 | 7 |
| K1 | .601 | .413 | -.304 | -.376 | .171 | .179 | -.170 |
| K2 | .365 | .430 | -.103 | .044 | -.196 | -.032 | .085 |
| K3 | .328 | .393 | -.141 | .037 | -.103 | -.114 | .126 |
| K4 | .499 | .317 | -.172 | -.136 | .090 | .094 | .035 |
| K5 | .475 | .403 | -.281 | -.154 | -.021 | .048 | -.108 |
| K6 | .490 | .267 | -.283 | -.408 | .134 | .147 | -.223 |
| K7 | .453 | .398 | -.155 | .045 | -.151 | -.060 | -.007 |
| K8 | .361 | .165 | -.205 | .219 | .197 | -.311 | .095 |
| K9 | .450 | .289 | -.188 | .322 | -.018 | -.338 | .063 |
| K10 | .473 | .309 | -.175 | .168 | .011 | -.317 | .074 |
| K11 | .213 | .182 | .064 | .052 | .074 | .040 | -.032 |
| A1 | .698 | -.186 | .069 | .034 | -.001 | .178 | -.010 |
| A2 | .680 | -.280 | .190 | .135 | -.129 | .180 | .071 |
| A3 | .739 | -.284 | .229 | .119 | -.100 | .136 | -.037 |
| A4 | .571 | -.311 | .187 | -.053 | .009 | .022 | .041 |
| A5 | -.136 | .405 | -.039 | .351 | -.317 | .199 | .068 |
| A6 | .562 | -.287 | .336 | .071 | -.040 | .009 | -.027 |
| A7 | .605 | -.342 | .242 | .047 | -.150 | -.026 | -.009 |
| A8 | -.146 | .431 | -.067 | .456 | -.204 | .293 | .119 |
| A9 | .448 | -.366 | .238 | -.133 | -.192 | -.119 | .015 |
| A10 | .441 | -.351 | .217 | -.028 | .205 | -.143 | .127 |
| P1 | -.015 | .239 | .299 | .404 | .143 | .016 | -.193 |
| P2 | -.001 | .276 | .319 | .425 | .160 | -.025 | -.251 |
| P3 | .020 | .206 | .310 | .378 | .309 | .032 | -.149 |
| P4 | .108 | .274 | .476 | .159 | .095 | -.034 | -.138 |
| P5 | .139 | -.206 | -.312 | .330 | .204 | .268 | .199 |
| P6 | .080 | -.322 | -.243 | .134 | .524 | .098 | .213 |
| P7 | .162 | -.195 | -.449 | .375 | .117 | .126 | .000 |
| P8 | -.073 | .461 | .402 | -.320 | .292 | -.002 | .165 |
| P9 | -.002 | .531 | .514 | -.213 | .088 | .113 | .247 |
| P10 | .072 | .475 | .412 | -.131 | -.017 | .087 | .199 |
| Extraction method: Principal Axis Factorisation. | | | | | | | |
| a. Seven factors were extracted. Twelve iterations were required. | | | | | | | |

**Supplementary table S2. Model fit indices of confirmatory factor analysis**

| Indicators | Reference | Actual |
| --- | --- | --- |
| CMIN/DF | 1-3: Excellent, 3-5: Good | 2.427 |
| RMSEA | <0.08: Good | 0.063 |
| IFI | >0.8: Good | 0.849 |
| TLI | >0.8: Good | 0.831 |
| CFI | >0.8: Good | 0.848 |

|  |  |  | **Estimate** | **Standardized Estimate** | **S.E.** | **C.R.** | **P** |
| --- | --- | --- | --- | --- | --- | --- | --- |
| K1 | <--- | Knowledge | 1 | 0.774 |  |  |  |
| K2 | <--- | Knowledge | 0.703 | 0.516 | 0.077 | 9.102 | <0.001 |
| K3 | <--- | Knowledge | 0.67 | 0.49 | 0.078 | 8.623 | <0.001 |
| K4 | <--- | Knowledge | 0.818 | 0.643 | 0.072 | 11.427 | <0.001 |
| K5 | <--- | Knowledge | 0.984 | 0.72 | 0.077 | 12.751 | <0.001 |
| K6 | <--- | Knowledge | 0.819 | 0.607 | 0.055 | 14.79 | <0.001 |
| K7 | <--- | Knowledge | 0.777 | 0.61 | 0.072 | 10.824 | <0.001 |
| K8 | <--- | Knowledge | 0.451 | 0.33 | 0.078 | 5.754 | <0.001 |
| K9 | <--- | Knowledge | 0.577 | 0.439 | 0.075 | 7.699 | <0.001 |
| K10 | <--- | Knowledge | 0.695 | 0.508 | 0.077 | 8.967 | <0.001 |
| K11 | <--- | Knowledge | 0.237 | 0.26 | 0.052 | 4.555 | <0.001 |
| A1 | <--- | Attitude | 1 | 0.716 |  |  | <0.001 |
| A2 | <--- | Attitude | 0.936 | 0.794 | 0.065 | 14.303 | <0.001 |
| A3 | <--- | Attitude | 1.002 | 0.847 | 0.066 | 15.195 | <0.001 |
| A4 | <--- | Attitude | 1.003 | 0.673 | 0.082 | 12.157 | <0.001 |
| A5 | <--- | Attitude | -0.4 | -0.218 | 0.102 | -3.936 | <0.001 |
| A6 | <--- | Attitude | 0.954 | 0.69 | 0.076 | 12.468 | <0.001 |
| A7 | <--- | Attitude | 1.025 | 0.732 | 0.078 | 13.222 | <0.001 |
| A8 | <--- | Attitude | -0.345 | -0.212 | 0.09 | -3.838 | <0.001 |
| A9 | <--- | Attitude | 0.859 | 0.571 | 0.083 | 10.329 | <0.001 |
| A10 | <--- | Attitude | 0.834 | 0.534 | 0.086 | 9.667 | <0.001 |
| P1 | <--- | Practice | 1 | 0.207 |  |  |  |
| P2 | <--- | Practice | 1.077 | 0.215 | 0.411 | 2.624 | 0.009 |
| P3 | <--- | Practice | 1.081 | 0.221 | 0.336 | 3.216 | 0.001 |
| P4 | <--- | Practice | 1.988 | 0.408 | 0.605 | 3.285 | 0.001 |
| P5 | <--- | Practice | -1.399 | -0.317 | 0.456 | -3.068 | 0.002 |
| P6 | <--- | Practice | -1.428 | -0.28 | 0.485 | -2.947 | 0.003 |
| P7 | <--- | Practice | -2.205 | -0.481 | 0.65 | -3.391 | <0.001 |
| P8 | <--- | Practice | 3.196 | 0.705 | 0.901 | 3.549 | <0.001 |
| P9 | <--- | Practice | 3.883 | 0.821 | 1.085 | 3.578 | <0.001 |
| P10 | <--- | Practice | 3.112 | 0.666 | 0.881 | 3.532 | <0.001 |

**Supplementary table 3. Distribution of knowledge dimension responses**

|  | **N (%)** | | |  |  |
| --- | --- | --- | --- | --- | --- |
|  | **True** | **False** | **Not sure** |  |  |
| **1. Chronic constipation may affect blood glucose control.** | 242(66.48%) | 2(0.55%) | 120(32.97%) |  |  |
| **2. Patients with diabetes should not increase their dietary fiber intake.** | 79(21.70%) | 199(54.67%) | 86(23.63%) |  |  |
| **3. Exercise is not helpful in relieving constipation.** | 114(31.32%) | 175(48.08%) | 75(20.60%) |  |  |
| **4.** **Patients with diabetes can prevent constipation by increasing fluid intake.** | 249(68.41%) | 21(5.77%) | 94(25.82%) |  |  |
| **5. Constipation is only related to dietary habits and has nothing to do with diabetes.** | 60(16.48%) | 191(52.47%) | 113(31.04%) |  |  |
| **6. Diabetes can lead to constipation.** | 213(58.52%) | 17(4.67%) | 134(36.81%) |  |  |
| **7. Patients with diabetes who experience constipation do not need to consult a doctor and can manage it on their own.** | 40(10.99%) | 248(68.13%) | 76(20.88%) |  |  |
|  | **A** | **B** | **C** | **D** | **E** |
| **9. Which of the following is not a common symptom of constipation?** | 34(9.34%) | 20(5.49%) | 184(50.55%) | 19(5.22%) | 107(29.40%) |
| **10. When patients with diabetes experience constipation, which method is not recommended for symptom relief?** | 38(10.44%) | 7(1.92%) | 232(63.74%) | 10(2.75%) | 77(21.15%) |
| **11. What is the primary cause of constipation in patients with diabetes?** | 44(12.09%) | 35(9.62%) | 8(2.20%) | 185(50.82%) | 92(25.27%) |
| **12. Which of the following medications is least likely to cause constipation?** | 11(3.02%) | 5(1.37%) | 10(2.75%) | 46(12.64%) | 292(80.22%) |
| **13. Constipation in patients with diabetes may be associated with which of the following factors?** |  |  |  |  |  |
| **A** | 268(73.63%) |  |  |  |  |
| **B** | 254(69.78%) |  |  |  |  |
| **C** | 202(55.49%) |  |  |  |  |
| **D** | 170(46.70%) |  |  |  |  |
| **E** | 84(23.08%) |  |  |  |  |
| **14. Under which of the following circumstances should patients with diabetes seek medical attention for constipation?** |  |  |  |  |  |
| **A** | 259(71.15%) |  |  |  |  |
| **B** | 241(66.21%) |  |  |  |  |
| **C** | 223(61.26%) |  |  |  |  |
| **D** | 200(54.95%) |  |  |  |  |
| **E** | 87(23.90%) |  |  |  |  |

**Supplementary table 4. Distribution of attitude dimension responses**

|  | N (%) | | | | |
| --- | --- | --- | --- | --- | --- |
|  | Strongly agree | Agree | Neutral | Disagree | Strongly disagree |
| 1. I believe constipation affects blood glucose control in patients with diabetes. | 132(36.26%) | 161(44.23%) | 46(12.64%) | 11(3.02%) | 14(3.85%) |
| 2. If I experience symptoms of constipation, I am willing to change my dietary habits to relieve it. | 150(41.21%) | 173(47.53%) | 28(7.69%) | 5(1.37%) | 8(2.20%) |
| 3. I believe regular exercise is very important for relieving constipation. | 150(41.21%) | 173(47.53%) | 28(7.69%) | 5(1.37%) | 8(2.20%) |
| 4. I believe patients with diabetes should avoid using laxatives. | 105(28.85%) | 158(43.41%) | 52(14.29%) | 39(10.71%) | 10(2.75%) |
| 5. I believe constipation does not significantly affect my daily life. | 51(14.01%) | 85(23.35%) | 56(15.38%) | 125(34.34%) | 47(12.91%) |
| 6. I believe patients with diabetes should have regular bowel health check-ups. | 114(31.32%) | 157(43.13%) | 67(18.41%) | 15(4.12%) | 11(3.02%) |
| 7. If I experience constipation, I am willing to try non-pharmacological treatment methods to relieve it. | 104(28.57%) | 172(47.25%) | 57(15.66%) | 18(4.95%) | 13(3.57%) |
| 8. I believe constipation is a difficult topic to talk about. | 32(8.79%) | 77(21.15%) | 91(25.00%) | 126(34.62%) | 38(10.44%) |
| 9. Persistent constipation related to diabetes makes me feel depressed. | 78(21.43%) | 155(42.58%) | 73(20.05%) | 46(12.64%) | 12(3.30%) |
| 10. If I experience constipation, I am willing to try treatments such as acupuncture and other traditional therapies. | 71(19.51%) | 139(38.19%) | 90(24.73%) | 46(12.64%) | 18(4.95%) |

**Supplementary table 5. Distribution of practice dimension responses**

|  | N (%) | | | | |
| --- | --- | --- | --- | --- | --- |
|  | Always | Often | Sometimes | Rarely | Never |
| 1. I regularly consume enough dietary fiber to prevent constipation. | 26(7.14%) | 99(27.20%) | 94(25.82%) | 110(30.22%) | 35(9.62%) |
| 2. I have a regular daily bowel movement schedule. | 24(6.59%) | 65(17.86%) | 82(22.53%) | 128(35.16%) | 65(17.86%) |
| 3. I engage in aerobic exercise at least three times a week. | 23(6.32%) | 100(27.47%) | 78(21.43%) | 117(32.14%) | 46(12.64%) |
| 4. When I experience constipation, I actively seek help from a doctor. | 35(9.62%) | 122(33.52%) | 110(30.22%) | 61(16.76%) | 36(9.89%) |
| 5. When I feel the urge to defecate, I delay going to the restroom. | 99(27.20%) | 129(35.44%) | 94(25.82%) | 33(9.07%) | 9(2.47%) |
| 6. I sit for long periods in my daily work and life. | 43(11.81%) | 115(31.59%) | 84(23.08%) | 90(24.73%) | 32(8.79%) |
| 7. When I experience constipation, I take laxatives or heat-clearing medications on my own. | 147(40.38%) | 110(30.22%) | 77(21.15%) | 18(4.95%) | 12(3.30%) |
| 8. When I experience constipation, I try exercises like Tai Chi or Baduanjin to help improve bowel function. | 139(38.19%) | 113(31.04%) | 77(21.15%) | 27(7.42%) | 8(2.20%) |
| 9. When I experience constipation, I try abdominal massage or other manual therapy techniques to relieve it. | 92(25.27%) | 115(31.59%) | 102(28.02%) | 42(11.54%) | 13(3.57%) |
| 10. When I experience constipation, I search for relief methods through media platforms, newspapers, books, etc. | 101(27.75%) | 116(31.87%) | 101(27.75%) | 33(9.07%) | 13(3.57%) |

**Supplementary table 6. SEM model fit**

| Indicators | Referenc | Measured results |
| --- | --- | --- |
| CMIN/DF | 1-3 excellent，3-5 good | 2.575 |
| IFI | >0.8 good | 0.832 |
| TLI | >0.8 good | 0.813 |
| CFI | >0.8 good | 0.831 |
| RMSEA | <0.08 Good | 0.066 |
| SRMR | <0.08 Good | 0.080 |

**Supplementary table 7. Estimated total effect coefficient**

| **Path** | | | **Estimate** | **S.E.** | **C.R.** | **P** |
| --- | --- | --- | --- | --- | --- | --- |
| Attitude | <--- | Knowledge | 0.49 | 0.108 | 4.547 | *** |
| Practice | <--- | Attitude | -0.073 | 0.033 | -2.235 | 0.025 |
| Practice | <--- | Knowledge | 0.149 | 0.06 | 2.504 | 0.012 |
| K1 | <--- | Knowledge | 1 |  |  |  |
| K2 | <--- | Knowledge | 0.78 | 0.08 | 9.733 | *** |
| K3 | <--- | Knowledge | 0.752 | 0.08 | 9.343 | *** |
| K4 | <--- | Knowledge | 0.834 | 0.075 | 11.12 | *** |
| K5 | <--- | Knowledge | 1.002 | 0.081 | 12.384 | *** |
| K6 | <--- | Knowledge | 0.809 | 0.057 | 14.312 | *** |
| K7 | <--- | Knowledge | 0.816 | 0.075 | 10.862 | *** |
| K8 | <--- | Knowledge | 0.472 | 0.081 | 5.817 | *** |
| K9 | <--- | Knowledge | 0.616 | 0.078 | 7.918 | *** |
| K10 | <--- | Knowledge | 0.728 | 0.081 | 9.022 | *** |
| K11 | <--- | Knowledge | 0.242 | 0.054 | 4.513 | *** |
| A10 | <--- | Attitude | 1 |  |  |  |
| A9 | <--- | Attitude | 1.03 | 0.122 | 8.422 | *** |
| A8 | <--- | Attitude | -0.412 | 0.111 | -3.699 | *** |
| A7 | <--- | Attitude | 1.23 | 0.126 | 9.787 | *** |
| A6 | <--- | Attitude | 1.144 | 0.121 | 9.467 | *** |
| A5 | <--- | Attitude | -0.478 | 0.126 | -3.79 | *** |
| A4 | <--- | Attitude | 1.203 | 0.129 | 9.328 | *** |
| A3 | <--- | Attitude | 1.202 | 0.114 | 10.513 | *** |
| A2 | <--- | Attitude | 1.124 | 0.11 | 10.204 | *** |
| A1 | <--- | Attitude | 1.199 | 0.124 | 9.662 | *** |
| P1 | <--- | Practice | 1 |  |  |  |
| P2 | <--- | Practice | 1.096 | 0.425 | 2.579 | 0.01 |
| P3 | <--- | Practice | 1.089 | 0.331 | 3.294 | *** |
| P4 | <--- | Practice | 2.024 | 0.627 | 3.228 | 0.001 |
| P5 | <--- | Practice | -1.546 | 0.501 | -3.087 | 0.002 |
| P6 | <--- | Practice | -1.468 | 0.504 | -2.913 | 0.004 |
| P7 | <--- | Practice | -2.318 | 0.693 | -3.345 | *** |
| P8 | <--- | Practice | 3.28 | 0.942 | 3.481 | *** |
| P9 | <--- | Practice | 3.931 | 1.121 | 3.506 | *** |
| P10 | <--- | Practice | 3.157 | 0.912 | 3.461 | *** |
