# Supplementary material for: Knowledge, attitudes, and practices regarding constipation among patients with type 2 diabetes mellitus: a structural equation modeling analysis
Source: Front Public Health. 2026 Mar 10;14:1728483. doi: 10.3389/fpubh.2026.1728483 (PMC13008887; doi:10.3389/fpubh.2026.1728483)
Supplement: Supplementary file 4 [file Data_Sheet_3.docx]

| Questionnaire ID： | | |  |
| --- | --- | --- | --- |
| Dear Participant,  We are researchers from Gansu Provincial People's Hospital, and we sincerely invite you to participate in our research project. This study aims to understand the knowledge, attitudes, and practices (KAP) of patients with type 2 diabetes mellitus (T2DM) regarding constipation, in order to provide a basis for developing scientifically sound intervention strategies. This may help more people in the future and improve their health condition. Your participation in this study is completely voluntary. The study has been approved by the Ethics Review Committee. If you agree to participate, please refer to the following instructions:  1. Please complete the questionnaire. There are no right or wrong answers; simply respond based on your actual situation. If you have any questions during the process, feel free to ask us. Once completed, please submit the questionnaire promptly.  2. This study involves a simple questionnaire survey and will not cause any harm to your physical or mental health. However, it may involve some personal information, such as your gender and age. Please rest assured that all your information will be kept strictly confidential and will not be disclosed.  3. As a participant, you may inquire about any information or research progress related to this study at any time. If you decide to withdraw from the study, please notify us. Your data will not be included in the final results.  Finally, we sincerely thank you for taking the time out of your busy schedule to support our scientific research！  □I have read and agree to allow the collected data to be used for scientific research.  Informed Consent Signature:  Date of Participation: Year Month Day | | |  |
|  | | |  |
| **Part 1 Basic Information** | | | |
| **1. Your gender:** | | A. Male | B. Female |
| **2. Your age: .** | | | |
| **3.** **Your height: cm.** | | | |
| **4. Your weight: kg.** | | | |
| **5. Your Body Mass Index (BMI = weight (kg) / height² (m²)):** | | A. Normal (18.5–23.9) B. Overweight (24–27.9) C. Obese (≥28) | |
| **6. Your average monthly income:** | | A. Low income (less than 2,000 RMB) B. Lower-middle income (2,001–5,000 RMB) C. Upper-middle income (5,001–13,000 RMB) D. High income (above 13,000 RMB) | |
| **7. Your education level:** | | A. Primary school or below B. Junior high school C. Senior high school/Technical secondary school D. Associate/Bachelor’s degree or above | |
| **8. Marital status:** | | A. Unmarried  B. Married  C. Divorced  D. Widowed | |
| **9. Are you a vegetarian?** | | A. Yes  B. No | |
| **10. Duration since your diagnosis of type 2 diabetes mellitus (T2DM):** | | A. ≤5 years  B. 6–10 years  C. 11–20 years  D. ≥20 years | |
| **11. Is your current blood glucose level under control?** | | A. Yes  B. No | |
| **12. Do you smoke?** | | A. Yes  B. No | |
| **13. Do you drink alcohol?** | | A. Yes  B. No | |
| **14. In the past six months, have you experienced any of the following defecation-related issues?** | | A. Straining during bowel movements, hard stools, a feeling of incomplete evacuation, sensation of anorectal obstruction, or needing manual assistance B. Fewer than 3 spontaneous bowel movements per week C. Diagnosed with constipation by a physician D. None of the above | |

**Part 2 Knowledge Dimension of Constipation Among Patients with Type 2 Diabetes Mellitus**

**Please select the option that best reflects your understanding of each question. If you are unsure, choose “Uncertain.”**

| **True/False Questions:** | |
| --- | --- |
| 1. Chronic constipation may affect blood glucose control. | 1. True B. False C. Not sure |
| 1. Patients with diabetes should not increase their dietary fiber intake. | 1. True B. False C. Not sure |
| 1. Exercise is not helpful in relieving constipation. | 1. True B. False C. Not sure |
| 1. Patients with diabetes can prevent constipation by increasing fluid intake. | 1. True B. False C. Not sure |
| 1. Constipation is only related to dietary habits and has nothing to do with diabetes. | 1. True B. False C. Not sure |
| 1. Diabetes can lead to constipation. | 1. True B. False C. Not sure |
| 1. Patients with diabetes who experience constipation do not need to consult a doctor and can manage it on their own. | 1. True B. False C. Not sure |
| 1. Chronic constipation may affect blood glucose control. | 1. True B. False C. Not sure |
| **Single-Choice Questions:** | |
| 1. Which of the following is not a common symptom of constipation? | A. Reduced bowel movement frequency B. Difficulty in defecation C. Frequent urination D. Hard stools E. Uncertain |
| 1. When patients with diabetes experience constipation, which method is not recommended for symptom relief? | A. Increasing dietary fiber intake B. Increasing fluid intake C. Arbitrary use of laxatives D. Regular physical activity E. Uncertain |
| 1. What is the primary cause of constipation in patients with diabetes? | A. Poor dietary habits B. Lack of physical activity C. Side effects of medications D. All of the above E. Uncertain |
| 1. Which of the following medications is least likely to cause constipation? | A. Antidepressants B. Antiepileptic drugs C. Antihypertensive drugs D. Insulin E. Uncertain |
| **Multiple-Choice Questions:** | |
| 1. Constipation in patients with diabetes may be associated with which of the following factors? (Select all that apply) | A. Lack of dietary fiber B. Insufficient physical activity C. Long-term use of certain medications D. Diabetic neuropathy E. Uncertain |
| 1. Under which of the following circumstances should patients with diabetes seek medical attention for constipation? (Select all that apply) | A. Constipation symptoms progressively worsen B. Accompanied by severe abdominal pain C. Presence of hematochezia or melena D. Significant weight loss E. Uncertain |

| **Part 3 Attitude Dimension Toward Constipation Among Patients with Type 2 Diabetes Mellitus**  Please indicate the extent to which you agree with the following statements by selecting one option from “Strongly Agree” to “Strongly Disagree.” | | | | | | | | |
| --- | --- | --- | --- | --- | --- | --- | --- | --- |
| 1. I believe constipation affects blood glucose control in patients with diabetes. | a. strongly agree | | | b. agree | c. neutral | d. disagree | e. strongly disagree | |
| 1. If I experience symptoms of constipation, I am willing to change my dietary habits to relieve it. | a. strongly agree | | | b. agree | c. neutral | d. disagree | e. strongly disagree | |
| 1. I believe regular exercise is very important for relieving constipation. | a. strongly agree | | | b. agree | c. neutral | d. disagree | e. strongly disagree | |
| 1. I believe patients with diabetes should avoid using laxatives. | a. strongly agree | | | b. agree | c. neutral | d. disagree | e. strongly disagree | |
| 1. I believe constipation does not significantly affect my daily life. | a. strongly agree | | | b. agree | c. neutral | d. disagree | e. strongly disagree | |
| 1. I believe patients with diabetes should have regular bowel health check-ups. | a. strongly agree | | | b. agree | c. neutral | d. disagree | e. strongly disagree | |
| 1. If I experience constipation, I am willing to try non-pharmacological treatment methods to relieve it. | a. strongly agree | | | b. agree | c. neutral | d. disagree | e. strongly disagree | |
| 1. I believe constipation is a difficult topic to talk about. | a. strongly agree | | | b. agree | c. neutral | d. disagree | e. strongly disagree | |
| 1. Persistent constipation related to diabetes makes me feel depressed. | a. strongly agree | | | b. agree | c. neutral | d. disagree | e. strongly disagree | |
| 1. If I experience constipation, I am willing to try treatments such as acupuncture and other traditional therapies. | a. strongly agree | | | b. agree | c. neutral | d. disagree | e. strongly disagree | |
| **Part 4 Practice Dimension Toward Constipation Among Patients with Type 2 Diabetes Mellitus**  Please indicate how often you engage in the following behaviors by selecting one option from “Never” to “Always.” | | | | | | | |  |
| 1. I regularly consume enough dietary fiber to prevent constipation. | | a. never | | b. rarely | c. sometimes | d. often | e. always |  |
| 1. I have a regular daily bowel movement schedule. | | a. never | | b. rarely | c. sometimes | d. often | e. always |  |
| 1. I engage in aerobic exercise at least three times a week. | | a. never | | b. rarely | c. sometimes | d. often | e. always |  |
| 1. When I experience constipation, I actively seek help from a doctor. | | a. never | | b. rarely | c. sometimes | d. often | e. always |  |
| 1. When I feel the urge to defecate, I delay going to the restroom. | | a. never | | b. rarely | c. sometimes | d. often | e. always |  |
| 1. I sit for long periods in my daily work and life. | | a. never | | b. rarely | c. sometimes | d. often | e. always |  |
| 1. When I experience constipation, I take laxatives or heat-clearing medications on my own. | | | a. never | b. rarely | c. sometimes | d. often | e. always |  |
| 1. When I experience constipation, I try exercises like Tai Chi or Baduanjin to help improve bowel function. | | | a. never | b. rarely | c. sometimes | d. often | e. always |  |
| 1. When I experience constipation, I try abdominal massage or other manual therapy techniques to relieve it. | | | a. never | b. rarely | c. sometimes | d. often | e. always |  |
| 1. When I experience constipation, I search for relief methods through media platforms, newspapers, books, etc. | | | a. never | b. rarely | c. sometimes | d. often | e. always |  |
